# Phytochemicals as an Alternative or Integrative Option, in Conjunction with Conventional Treatments for Hepatocellular Carcinoma

**DOI:** 10.3390/cancers13225753

**Published:** 2021-11-17

**Authors:** Sheryl Rodriguez, Kristy Skeet, Tugba Mehmetoglu-Gurbuz, Madeline Goldfarb, Shri Karri, Jackelyn Rocha, Mark Shahinian, Abdallah Yazadi, Seeta Poudel, Ramadevi Subramani

**Affiliations:** 1 Center of Emphasis in Cancer Research, Department of Molecular and Translational Medicine, Paul L. Foster School of Medicine, Texas Tech University Health Sciences Center El Paso, El Paso, TX 79905, USA; sheryl.rodriguez@ttuhsc.edu (S.R.); tugba.gurbuz@ttuhsc.edu (T.M.-G.); sepoudel@ttuhsc.edu (S.P.); 2 Graduate School of Biomedical Sciences, Texas Tech University Health Sciences Center, El Paso, TX 79905, USA; kristy.skeet@ttuhsc.edu (K.S.); jackie.rocha@ttuhsc.edu (J.R.); mark.shahinian@ttuhsc.edu (M.S.); ayazadi@ttuhsc.edu (A.Y.); 3 Paul L. Foster School of Medicine, Texas Tech University Health Sciences Center El Paso, El Paso, TX 79905, USA; madeline.goldfarb@ttuhsc.edu (M.G.); shri.karri@ttuhsc.edu (S.K.)

**Keywords:** hepatocellular carcinoma, liver cancer, phytochemicals, natural compounds, metastasis

## Abstract

**Simple Summary:**

Hepatocellular carcinoma (HCC) is globally ranked as the sixth most diagnosed cancer, and the second most deadly cancer. To worsen matters, there are only limited therapeutic options currently available; therefore, it is necessary to find a reservoir from which new HCC treatments may be acquired. The field of phytomedicine may be the solution to this problem, as it offers an abundance of plant-derived molecules, which show capabilities of being effective against HCC proliferation, invasion, migration, and metastasis. In our review, we collect and analyze current evidence regarding these promising phytochemical effects on HCC, and delve into their potential as future chemotherapies. Additionally, information on the signaling behind these numerous phytochemicals is provided, in an attempt to understand their mechanisms. This review makes accessible the current body of knowledge pertaining to phytochemicals as HCC treatments, in order to serve as a reference and inspiration for further research into this subject.

**Abstract:**

Hepatocellular carcinoma (HCC) is the most abundant form of liver cancer. It accounts for 75–85% of liver cancer cases and, though it ranks globally as the sixth most common cancer, it ranks second in cancer-related mortality. Deaths from HCC are usually due to metastatic spread of the cancer. Unfortunately, there are many challenges and limitations with the latest HCC therapies and medications, making it difficult for patients to receive life-prolonging care. As there is clearly a high demand for alternative therapy options for HCC, it is prudent to turn to plants for the solution, as their phytochemicals have long been used and revered for their many medicinal purposes. This review explores the promising phytochemical compounds identified from pre-clinical and clinical trials being used either independently or in conjunction with already existing cancer therapy treatments. The phytochemicals discussed in this review were classified into several categories: lipids, polyphenols, alkaloids, polysaccharides, whole extracts, and phytochemical combinations. Almost 80% of the compounds failed to progress into clinical studies due to lack of information regarding the toxicity to normal cells and bioavailability. Although large obstacles remain, phytochemicals can be used either as an alternative or integrative therapy in conjunction with existing HCC chemotherapies. In conclusion, phytochemicals have great potential as treatment options for hepatocellular carcinoma.

## 1. Introduction

Cancer is the second leading cause of death worldwide. According to the estimates of cancer incidence and mortality produced by the International Agency for Research on Cancer (GLOBOCAN 2020), liver cancer is ranked as the sixth most frequently diagnosed cancer, yet is ranked second in terms of mortality rate [1]. There are several types of liver cancers, which include hepatocellular carcinoma, intrahepatic cholangiocarcinoma, and hepatoblastoma. Among these subtypes, hepatocellular carcinoma (HCC) is the most prevalent [2], accounting for 75–85% of all cases of liver cancers diagnosed [3]. Due to the absence of effective treatment options, mortality rates for HCC are high, with a current 5-year survival rate of 8.37% [4]. Metastasis of cancerous cells to distal sites in the body is the leading cause of death in patients with HCC [5]. Hepatitis C virus (HCV) and hepatitis B virus (HBV) infections increase the risk of developing HCC [6,7]. HCC may also develop due to chronic liver diseases such as cirrhosis [8], fibrosis [9], or non-alcoholic fatty liver disease (NAFLD) [10]. Concerningly, these diseases are becoming more globally prevalent, leading to the expectation that HCC rates will only continue to climb in the future. High mortality rates paired with high incidence rates reflect the reality that HCC is quickly becoming a major global health problem.

## 2. HCC Treatments

There are currently very few surgical treatments available for HCC. Typically, early-stage HCC may be treated with surgical resection, liver ablation, or liver transplantation. However, each of these treatment options has its own obstacles and drawbacks [11]. Further, once extrahepatic spread has occurred, these therapies drop in efficacy. For this reason, few HCC patients qualify for any of the aforementioned treatments, and, for those who do qualify, recurrence of the cancer is a legitimate concern and possibility [12,13,14].

Pharmaceutically, there are also few systemic drug treatment options. Multi-kinase inhibitors, such as sorafenib and lenvatinib, are generally the first-line systematic treatments given to patients with cases of unresectable HCC [15,16]. However, these drugs are not characterized as curative, and are administered either as an attempt to control the cancer progression so that surgery is possible, or as an attempt to increase the life expectancy; in many cases, by only a few months [17]. Additionally, it is well known that patients develop resistance to these drugs over time [18,19,20], resulting in short-lived efficacy. Furthermore, due to these treatments, patients suffer from untargeted tissue damage and side effects such as hair loss, low red blood cell count, GI tract infection, and immunosuppression. Currently, only limited treatment options are available to treat HCC and even those treatments are not highly effective. Hence, the patients who do not respond to any of the treatments have no other choice except palliative care [21,22,23].

### Phytochemicals in Hepatocellular Carcinoma Treatment and Prevention

Historically, natural products have played a dominant role in the evolution of sophisticated traditional medicine, with available records showing natural products having been used for medicine as far back as 2900 BCE [24]. One of the best-documented examples is the ancient Egyptian record *Ebers Papyrus*, which lists over 700 plant-based compounds that were used for medicinal purposes [25]. As our ancestors recognized long ago, nature is a rich source of medicines, which could treat many ailments.

**Figure 1:**
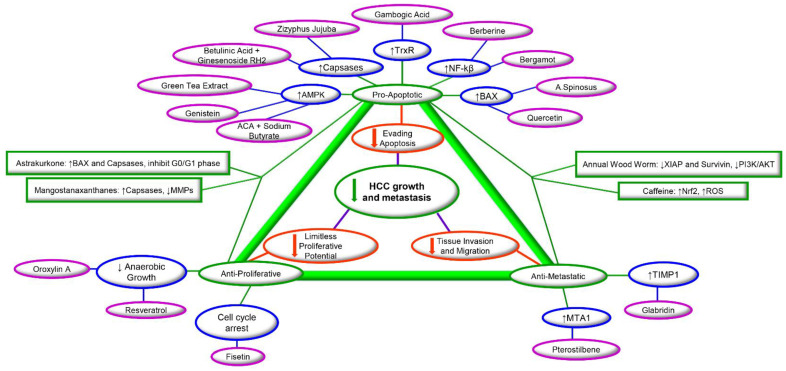
Phytochemicals and their mechanisms to target HCC metastasis are shown here. Phytochemicals effect the major processes of HCC metastasis, such as cell cycle, proliferation, migration, invasion, and apoptosis by targeting key molecular markers involved in these processes.

Although herbal medicines are still being used extensively around the world, there persists a vast lag in the acceptance of their validity by the scientific and medical community due to the lack of scientific evidence. Further study into these compounds is much needed to help bridge the current gap between pre-clinical research and clinical application, as plants are rich in secondary metabolites that have been previously shown to be potent chemotherapeutic and/or chemopreventive agents used for numerous chronic cardiovascular, metabolic, neurodegenerative, and neoplastic diseases [26,27,28]. Although there is promising evidence, many phytochemicals lack the proper concrete scientific soundness to be able to be safely carried on into further levels of research, simply because they currently have not been studied enough. Despite these obstacles involved in successful clinical translation, many phytochemicals have fueled much progress in the field of cancer treatment including HCC. Dietary phytochemicals, such as curcumin [29,30], resveratrol [31,32], quercetin [33], silybin [34], N-trans-feruloyl octopamine [35], lycopene [36,37,38], emodin [39,40], caffeine [41], and phloretin [42,43], have been shown to possess anti-cancer properties against HCC. Phytochemicals are effective in attenuating the main hallmarks of cancer such as proliferation, migration, and invasion, and are capable of inducing apoptosis to control HCC metastasis through targeting key molecular markers involved in these processes (Figure 1). Additionally, according to earlier reports, 60% of the anti-cancer medication in current use has been obtained from natural sources [35]. In fact, natural sources present close to 50% of all new chemical entities approved from 2000–2006 [44].

While some research has been conducted to provide evidence for the efficacy of treatments derived from plant sources, there is more progress to be made, as the need for alternative or integrative options in cancer treatment is evident. Natural plant-derived products would provide a wide availability of options for the testing of efficient treatments for HCC. Such products are innately abundant in nature, and research to date has found that they lead to fewer and less toxic side effects [45]. If medicine (in the field of cancer) is to continue to evolve into the future, further exploration and understanding into the properties and potentials of phytochemicals is required.

## 3. Types of Phytochemicals with Anticancer Activity against HCC

**Figure 2:**
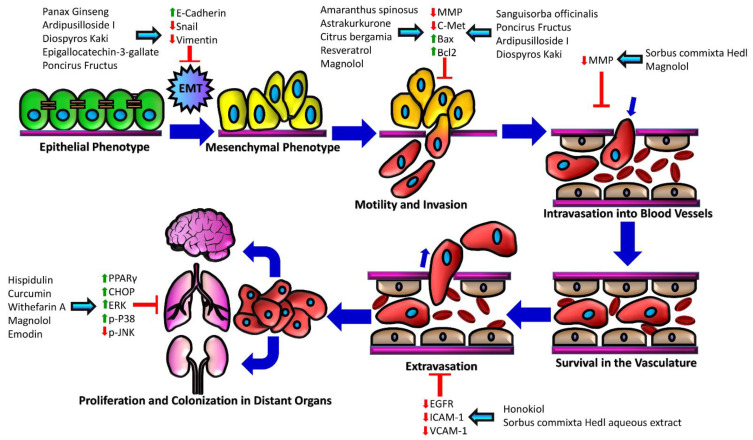
Phytochemicals target crucial molecular signaling markers involved in various key processes of HCC metastasis. Several phytochemicals inhibit specific stages of HCC metastasis, such as epithelial–mesenchymal transition (EMT), motility, invasion, intravasation, extravasation, and colonization in distant organs.

Several natural compounds derived from plant sources have been evaluated for their effectiveness as potential treatment options against hepatocellular carcinoma (Table 1). Furthermore, there are numerous phytochemicals tested in clinical trials for HCC treatment, which shows hope for the future of phytochemicals as a therapeutic agent for HCC (Table 2). Preclinical studies on major classes of bioactive molecules are being conducted and they are producing promising results. Even more promising is the fact that, several phytochemicals show evidence in being effective against the various stages of metastasis, which is the main cause of death for those with HCC (Figure 2). Here, we discuss the anti-cancer effects of several major categories of phytochemicals on HCC. Studies discussed in this review were obtained thorough searches of PubMed database using combinations of the terms “hepatocellular carcinoma”, “HCC”, “liver cancer”, “phytochemical”, “plant-based medicine”, “natural compounds”, “biomolecules”, and “plant-based compounds”.

### 3.1. Lipids

Lipids are a class of biochemical compounds, made up primarily of fatty acids. Different lipids and their derivatives from plants have been shown to have anti-cancer potential against HCC. *Amaranthus spinosus*, otherwise known as spiny amaranth, is a plant native to warm, tropical climates, including tropical Americas. It is considered an invasive weed in certain environments, but a leafy vegetable in others [132], and has been used medicinally in the Sub-Himalayan region of India [133]. Studies have shown its potential medicinal use for jaundice [133], diabetes [134], diuresis [135], gastrointestinal constipation [136], and bacterial infections [137]. Until recently, few studies had been published regarding its anti-cancer properties against hepatocellular carcinoma. In a study, (14E, 18E, 22E, 26E)-methyl nonacosa-14, 18, 22, 26 tetraenoate, a fatty acid isolated from *A. spinosus*, first demonstrated its potent ability to inhibit HepG2 HCC cell proliferation and apoptosis via upregulation of Bax and downregulation of Bcl-2 and cyclin B1, when administered at a dose of 25.52 µmol/L [46]. The IC_50_ of doxorubicin for the same cell line was also found by this study to be 24.68 µmol/L. This suggests that the dosage for the fatty acid from *Amaranthus spinosus* is feasible to attain in real-world treatments, as it is comparable to the dosage of currently used chemotherapy doxorubicin.

Lipiodol (an oil derived from poppy seeds) contains 38% iodine by weight [138], and is cleared from HCC cells less rapidly than it is cleared from normal hepatocytes. This compound can also be made to be radioactive, resulting in iodine-131-labeled lipiodol. These characteristics of lipiodol allows the radioactive treatment of iodine-l31-labeled lipiodol to be used as an HCC treatment tool. When administered to patients, it allows cancerous liver tissues to receive about an eight times stronger radiation dose than normal liver tissue, making for an ideal way to target the specific cancerous tissues within patients [125,138]. Although originally used as a contrast medium for radiology use, several clinical trials have found success in using iodine-131-labeled lipiodol as a treatment capable of increasing overall survival for patients with HCC [125,126,127]. This treatment was found to be well tolerated and even effective in small tumors [125]. Lipiodol is a good example of a phytochemical which helps provide a pathway to HCC treatment, despite it being used only as a carrier for radiation. It is important to note that phytochemicals do not necessarily have to outright kill cancer cells to still be able to be used to combat cancer through different mechanisms.

#### Terpenoids

Terpenoids are non-saponifiable lipids or simple lipids found in many plants and mushrooms across the world. They are made up of differing numbers of linked isoprene units, and may be classified as monoterpenes, diterpenes, triterpenes, sesquiterpenes, etc., depending on the number of isoprene units present in the molecule [139,140,141,142]. Terpenoids are one of the largest classes of phytochemicals [143] that have shown to have anti-cancer effects against various cancers, including hepatocellular carcinoma [144,145]. Mushrooms are especially rich in terpenoids [145], and their extracts are currently being explored as potential pharmaceuticals [146], with some mushrooms already being used in the clinical setting [146,147,148]. Astrakurkurone (a triterpene isolated from the edible mushroom *Astraeus hygrometricus*) has demonstrated potential for its use as a cytotoxic agent against HCC cell lines (Hep3B and HepG2) at distinctively low doses (LD50 of 58.8 µM and 122 µM respectively) [47]. Toxicity was also found to be selective, as no cytotoxicity was found when administered to a normal liver cell line, even at the highest dose tested (250 µM). Astrakurkurone impeded cellular proliferation through cell cycle arrest at the sub-G0/G1 phase, and showed evidence of depolarizing the mitochondrial membrane through excessive ROS production and disruption of the mitochondrial membrane potential [47]. The compound also upregulated pro-apoptotic markers cleaved caspase-9 and Bax while downregulating the anti-apoptotic marker, Bcl-2. This study demonstrated that the triterpene astrakurkurone is a potential anti-cancer phytochemical for HCC treatment.

Fruits also contain terpenoids that demonstrate anti-cancer effects. *Ziziphus jujuba*, a fruit resembling a date, belongs to the *Rhamnaceae* family; it is grown in Asia, Australia, Europe, and Mediterranean regions. This fruit contains various bioactive compounds, including many different triterpenic acids [149]. A previous study by Kim et al., indicated that ursolic acid, one of *Ziziphus jujuba’s* triterpenic acids, decreased cell viability and exhibited pro-apoptotic activity against HepG2 cells (when given at a dose of 30 µM) [48]. Although the exact mechanism is yet to be elucidated, it is suggested that ursolic acid (UA) is able to induce cell cycle arrest through disruption of DNA fork establishment during the initiation stage of the cell cycle [48]. Moreover, UA causes increased expression of p21/WAF1, which may also cause cell-cycle arrest and induce apoptosis through release of cytochrome c and caspase-3 activation [48]. Another study also demonstrated the anti-tumor effects of UA using Huh-7 HCC cells [50]. Oleanolic acid (OA), another triterpenic acid, along with UA were shown to induce apoptosis (at a dose of 20 µM) by acting on the permeability of the mitochondria, leading to the release of cytochrome C and activation of pro-apoptotic markers caspase-9, caspase-3, and PARP. The study also showed that OA and UA could inhibit the expression of X-linked inhibitors of apoptotic protein (XIAP) mRNA, which is typically elevated in cancer cells [50]. In addition, when OA was subcutaneously injected (1.0 g/kg) to mice or rats, it did not cause any toxicity or mortality [150]. Furthermore, after oral administration of OA (at 180 mg/kg) to mice for 10 days, no abnormalities were observed in brain, heart, liver, kidney, thyroid, testes, stomach, intestine, or spleen [150]. OA and UA have also been shown to induce apoptosis in a dose-dependent (2–8 µmol/L) manner in various human liver cancer cell lines through increase of DNA fragmentation, lowering of Na+-K+-ATPase activity, decrease of mitochondrial membrane potential, elevation of caspase-3 and caspase-8 activity, and suppression of the production of angiogenic signaling protein VEGF and cell adhesion molecule ICAM-1 [151]. Effectivity at such low dosages is a promising characteristic, which could allow an easier and safer transition to clinical practice. The smaller the dose needed, the more feasible it may be to accomplish such dosing level in humans.

Diol-type ginsenosides are another major triterpenoid isolated from *Panax ginseng*; a traditional Chinese medicinal herb that has long been used as an anti-inflammatory, anti-cancer, and anti-pathogenic remedy [152]. Although *P. ginseng* is composed of many active components, one of these components (a ginsenoside) in particular, protopanaxadiol (PPD), was shown to be able to exert antioxidant and anti-inflammatory effects on HepG2 and PLC/PRF/5 hepatocellular carcinoma cells at 20 and 40 µM treatments [51]. Additionally, PPD has the potential to inhibit migration, invasion, and proliferation of these cancer cells by increasing the expression level of epithelial marker E-cadherin, and decreasing the expression level of mesenchymal marker vimentin. PPD can also exhibit its anti-cancer effects by targeting the STAT3/Twist1 pathway [51]. STAT3 is a key player in proliferation, migration, and angiogenesis. Typically, STAT3 is present in the cytoplasm in an inactive state until stimulated by cytokines, whereby it translocates to the nucleus and becomes active. PPD inhibits the translocation of STAT3 into the nucleus. Twist1, a target gene of STAT3, is also affected by PPD. Twist1 overexpression has been found to induce EMT of cancer cells. Furthermore, the inactivation of STAT3 by PPD hinders the ability of Twist1 to induce EMT to control metastasis [51]. PPD also has great potential to be used synergistically with existing chemotherapies. When intragastrically administered to cancer cell xenografted female BALB/C nude mice, the combination treatment of platinum anticancer drug oxaliplatin (OXA) with PPD (30 mg/kg PPD + 10 mg/kg OXA), was found to be significantly effective in suppressing tumor growth, when compared to treatment with PPD (30 mg/kg) or OXA (10 mg/kg) alone [51]. The ability to enhance the chemotherapeutic effects of existing anticancer medication is powerful, especially if the phytochemical has fewer side effects than the anticancer medication. This could allow administration of lesser doses of anticancer medication and thereby lead to fewer harmful side effects in patients. However, the research into what side effects may be caused by PPD administration is still quite far off. Despite this, the foundational promise PPD brings to the table is great.

Ardipusilloside I is a triterpene isolated from the Chinese medicinal herb *Ardisia pusilla* A. DC. This compound exerts anti-cancer effects against HCC cell lines HepG2 and SMMC-7721 by severely inhibiting their survival, invasion, and metastasis (when given at a concentration of 50 µM). These effects are believed to be accomplished through the suppression of the MEK/ERK and Akt signaling pathways, resulting in the downregulation of matrix metalloproteinase-2 (MMP-2) and matrix metalloproteinase-9 (MMP-9). Furthermore, ardipusilloside I has been shown to reduce metastasis of HCC cells by activating Rac1, which results in upregulation of E-cadherin, subsequently leading to reduced metastatic capabilities. In vivo experiments also exhibited the anti-cancer effects of Ardipusilloside I. Two groups of male athymic BALB/c nude mice were orthotopically implanted with HCC cells and treated with ardipusilloside I at two different doses (50 mg/kg/d and 100 mg/kg/d). Both treatments resulted in animals developing fewer metastatic tumors in their lungs compared to the untreated controls. This evidence suggests the ability of ardipusilloside I to negatively impact the metastatic capability of HCC [52]. Moreover, the tested doses in these animal models may be feasible to translate into humans. When moving forward from preclinical studies to clinical studies, the requirement of dose will usually decrease based on the animal’s body weight and metabolism [153]. As ardipusilloside I also has been shown to have non-toxic effects towards normal astrocytes [154], this compound shows great potential to become a therapeutic agent against HCC.

Lanatoside C or isolanid is a triterpene cardioglycoside extracted from the *Digitalis ferruginea* (namely “rusty foxglove”) plant. It has been shown that growth of PTEN deficient Mahlavu HCC cells was significantly reduced upon treatment with lanatoside C (at 2 µM) through induction of G2/M cell cycle arrest, JNK activation, induction of apoptosis, and ROS production in vitro. In addition, through MRI of nude mice (8–16 weeks old), lanatoside C was shown to reduce the size of HCC xenograft tumors in vivo by almost 50%, after administration via gavage feeding (6 mg/kg) for 21 days [53]. It is encouraging to see such effects in vivo after gavage administration, as this indicates the compound may be effective even when administered orally. Oral administration is safer compared to other forms, as it helps lower the risk of cytotoxicity [155,156]. Although much more research needs to be done, lanatoside C seems to have beneficial effects through what is generally considered a safer route of drug administration. Additionally, when administered to embryonic and normal hepatic cell lines (0.1 to 500 µM for 24 h), no toxicity was noted [157].

*Diospyros kaki* (also known as the kaki persimmon) has an abundance of phytochemicals to offer, as it is rich in terpenoids and flavonoids. Ethanol extracts from *D. kaki* leaves have been shown to regulate EMT and stemness features of HCC through inhibition of the hepatocyte growth factor (HGF)/Met pathway. HepG2, Hep3B, and SNU475 cell lines treated with ethanolic extract of *D. kaki* (50 µg/mL) indicated lower expression of CD44 and keratin19, as well as lower expression of snail, vimentin, MMP-1, and MMP-2/MMP-9. Simultaneously, an increased expression level of E-cadherin was observed after treatment with HGF+*D. kaki* leaf extract, suggesting *D. kaki* has the ability to inhibit cancer cell growth despite addition of HGF to the cells. Additionally, this study tested the effectiveness of gold standard chemotherapy treatment Sorafenib in conjunction with 50 µg/mL of *D. kaki*, and found that the IC_50_ of Sorafenib dropped from 3.7 µg/mL, 5.9 µg/mL, and 9.5 µg/mL in cancer cell lines Hep3B, HepG2, and SNU475 respectively, to 1.8 µg/mL, 2.7 µg/mL, and 5.9 µg/mL. Kaki leaf extract was not found to cause toxicity in cancer cells at concentrations up to 50 µg/mL, which was the maximum dose tested. Furthermore, a cytotoxicity test was performed on 5-week-old female ICR mice, to which oral doses of Kaki extract were given at 2 g/kg once daily for 14 days. The LD_50_ was unable to be determined from this experiment, as all mice survived all 14 days with no change in body weight when compared to controls. Additionally, when tested on normal human cell lines (MCF10A and HPL1A), no cytotoxicity was noted [158]. Although it is beneficial and promising that the extract was not found to be toxic to cells in vitro nor the whole organism in vivo, it may be jarring to hear that a prospective anti-cancer treatment has no cytotoxic effects on cancer cell lines. However, *D. kaki* extract does seem to have a net positive effect, as it does decrease the metastatic potential of the cancer cells [54]. Metastasis is a substantial potential complication in any cancer, and is the reason many patients do not survive for long after diagnosis, especially in the case of HCC. A phytochemical that shows impact on metastasis while not directly showing cytotoxicity towards cancer cells can still improve the lives and outcomes of many cancer patients.

*Sanguisorba officinalis* is a plant in the family of *Rosaceae*, which grows in the cooler regions of northern Europe. A triterpenoid saponin called Ziyuglycoside II (3β-3-α-1-arabinopyranosyloxy-19-hydroxyurs-12-en-28-oicacid) is found in the roots of this plant. Ziyuglycoside II is well known for its anti-cancer properties, including having an ameliorating effect on gastric carcinoma [159], breast cancer [160], and colon cancer [161]. Another study on Ziyuglycoside II indicated that it is also effective in impeding HCC growth in HepG2 and SMMC-7721 cancer cell lines through increasing ROS activity and inducing cell cycle arrest at G0/G1, which leads to apoptosis. Moreover, this compound was observed to decrease the migratory and invasive capabilities of these HCC cells in a dose-dependent (6.6 µg/mL–10 µg/mL) manner, by suppressing the expression of MMP-2 and MMP-9, as well as by inhibiting the EGFR/NF-kB pathway [55]. Additionally, Ziyuglycoside II has displayed a lowered level of toxicity to normal cells (BRL3A and HEK293T), indicating its cytotoxicity is selective to HCC [55].

*Brucea javanica* is a flowering shrub whose fruit has long been used in traditional Chinese medicine for the treatment of dysentery, malaria, and even cancer [162]. The fruit is very rich in quassinoids, which are triterpene lactones known for possessing significant anti-tumor activity. Brucea javanica oil (BJO) is obtained from extracts of the seeds of *Brucea javanica*. This oil contains many quassinoids, namely brusatol, which is one of the major quassinoids isolated from *B. javanica* [162]. Brusatol’s promising anti-cancer effects were reported in many cancer types including colorectal [162], lung [163], and pancreatic cancer [164]. Additionally, brusatol’s cytotoxic effect was also tested on normal gastric epithelial cells and found to be non-toxic [164]. When brusatol enriched BJO (BE-BJO) at 610 mg/kg, regular BJO (BJO) at 2000 mg/kg, and brusatol free BJO (BF-BJO) at 2000 mg/kg was injected intraperitoneally into male Kunming mice with implanted H22 cell hepatomas, it was found that tumor growth was suppressed in those mice treated with BE-BJO. Further, RNA analysis showed that BE-BJO significantly induced miRNA-29b and p53 expression. BJO and BE-BJO both downregulated Bcl-2 and enhanced the expression of Bax, Bad, cytosol cytochrome-c, cleaved caspase-3, cleaved caspase-9, and cleaved PARP to induce apoptosis of H22 xenografts in mice. Based on these findings, it is clear that brusatol (a major active compound from BJO) exerts significant HCC cell death [56]. Beneficially, it was also observed that administering 2000 mg/mL of each of BJO, BE-BJO, and BF-BJO orally for 14 days did not prove toxic to Kunming mice, as there were no deaths nor behavioral displays of toxicity. We speculate that the combination therapy of brusatol isolated from *Fructus Bruceae* given with existing therapies for HCC will improve patient life-span based on the effects observed in previous research conducted whereby cisplatin administered in the presence of brusatol demonstrated a synergistic therapeutic option for colon cancer [162].

Artemisinin is a sesquiterpene lactone compound found in sweet wormwood (*Artemisia annua*), a type of flowering herb that is native to temperate Asia and parts of North America. These herbs are often used in traditional Chinese medicine for the treatment of various illnesses, including fever and hemorrhoids, although the clinical potential of these flowering herbs extends much farther than their current use. Artemisinin compounds isolated from *Artemisia* have long displayed anti-cancer properties against various cancers [165], and these anti-cancer effects extend to HCC as well. Artemisinin derivatives were shown to inhibit HepG2 and Huh-7 cell proliferation (when given at 100 µg/mL) by blocking the PI3K/AKT and mTOR signaling pathways in these HCC cell lines. These pathways regulate both apoptosis and angiogenesis in HCC. Blocking such pathways controls the proliferative and angiogenic potential of HCC cells. Artemisinin was also shown to down-regulate the anti-apoptotic proteins XIAP and survivin, as well as increase the expression of cleaved caspase-3 and PARP. These actions result in increased apoptosis of Huh7 and HepG2 [57]. Additionally, tumor spheroids treated with artemisinin derivatives displayed tumor cell apoptosis and necrosis at rates greater than HCC control spheroids not treated with the artemisinin derivatives, indicating artemisinin’s effective cytotoxic potential against HCC. Further, artemisinin was found to be effective in inhibiting the invasive and migratory capabilities of HCC cells, as shown when HCC cells treated with artemisinin (at 12.5 µM) showed minimized motility compared to HCC control cells [58]. In vivo results also displayed the therapeutic potential of artemisinin. Male athymic BALB/c nude mice that were orthotopically implanted with HCC tumor tissues and then treated intragastrically with artemisinin daily (at 50 mg/kg and at 100 mg/kg for 4 weeks) were found to have fewer tumors that had metastasized to their lungs, compared to the untreated control mice. Additionally, when tested with a cell adhesion assay, artemisinin significantly increased the cell–cell adhesion of HCC cells, further indicating artemisinin’s prohibitive effects on the capability of HCC to metastasize [58].

Paclitaxel is a diterpenoid isolated from the Pacific Yew tree, which is currently used as a cytotoxic agent against solid tumors [128]. Its main method of action involves targeting dividing cells to induce cytotoxicity. Paclitaxel has shown activity that acts against tumors in lung, head, neck, and esophageal cancers as well as induced remissions [166]. This phytochemical has had success in clinical trials, and is now an FDA-approved treatment for breast cancer, lung cancer, ovarian cancer, and Kaposi sarcoma [166]. In one clinical trial exploring the potential to use paclitaxel for HCC treatment, there was found to be an improvement in tumor-related symptoms. More research is needed to explore the possibilities of using paclitaxel to treat HCC.

Ginsenoside Rg3 is a ginseng saponin obtained from the *Panax ginseng* plant that has been shown to attenuate signaling pathways regarding VEGF-dependent Akt/eNOS [167]. Moreover, ginsenoside Rg3 reduces phosphorylation of STAT3 resulting from hypoxic conditions, phosphorylation of ERK 1/2, JNK, and decreases VEGF expression [168]. Ginsenoside Rg3 was shown to inhibit microtumor vessel formation activation in mice when given at a dose of 10 mg/kg once a day and it contributed to tumor shrinkage and increased animal survival [169]. A study that assessed the pharmacokinetics of Ginsenoside Rg3 found that it was well tolerated by humans when administered intramuscularly at a dose ranging from 10–60 mg once every two days [170].

### 3.2. Phenols

Phenols make up a vast portion of all plant-produced secondary metabolites; over 8000 phenols have been identified in nature so far [171,172,173]. These phytochemicals are composed of an aromatic ring with one or more hydroxyl groups, and are found in a wide swath of plants, including various vegetables, fruits, and legumes [172,173]. Phenols may be further categorized into compounds, such as flavonoids, catechins, stilbenes, and xanthones [172,173].

#### 3.2.1. Flavonoids

Flavonoids are present in fruits, vegetables, grains, teas, and wines, making up a large and essential part of the human diet [174]. These compounds have been recognized for possessing anti-inflammatory, anti-oxidant, anti-bacterial, and anti-viral properties [174,175]. Unsurprisingly, various flavonoids have also shown potential as anti-cancer compounds.

*Citrus bergamia*, also known as bergamot, is a tree native to the Italian Calabria region. The juice from the citrus fruit of this tree is known to be largely composed of flavonoids, with the most abundant flavonoids in the juice being eriocitrin, neoeriocitrin, naringin, and neohesperidin [59]. When individually extracted and tested, these flavonoids have been shown to have anti-cancer effects on hepatocellular carcinoma cell lines [176,177,178]. Additionally, when tested on a normal liver cell line (L02) at doses up to 75 µM, eriocitrin caused low cytotoxicity [177]. Furthermore, as shown by Ferlazzo et al., the anticancer effect of these flavonoids persist even when the compounds are administered altogether. In the study, the treatment of HepG2 cells with 10% bergamot juice (which contains all the flavonoids mentioned above) decreased proliferation rate and induced pro-apoptotic effect. A total of 10% of bergamot juice induced cell cycle arrest in HepG2 cells in the G2 phase. This cell cycle arrest coincided with increased expression of p53 and p21 proteins, which may explain the mechanism by which cell cycle arrest occurred. Exposure to the flavonoids found in the bergamot juice also resulted in increased expression of apoptosis-related genes such as Bcl-2, caspase-8, caspase-9, caspase-3, PARP, TNF receptor (death effector domain), NF-kB, and IkB. Concurrently, anti-apoptotic genes (such as BIRC8 and Bcl-2L2) were found to be downregulated. NF-kB nuclear translocation was also found decreased. Hence, the flavonoids from bergamot juice may activate both mitochondrial intrinsic and Fas-mediated extrinsic apoptotic pathways [60].

Oroxylin A is another flavonoid, which has shown promise as a potential new treatment option for HCC. This flavonoid is isolated from the root of a mint-like plant called *Scutellariae radix* and has been used for thousands of years in Chinese medicine for various health conditions. Oroxylin A at 12.5, 25, and 50 µM concentrations was shown to reduce the generation of lactate and glucose in HepG2 cells under hypoxic conditions. It also inhibited the expression of HIF-1α, which is a key regulating protein of tumor cell energy metabolism under hypoxic conditions. This protein assists tumor cell survival in hypoxic and glycolytic conditions. Through suppression of HIF-1α, its downstream targets (PDK1, LDHA, and HK II) were also inhibited [61]. However, oroxylin A was also found to suppress ATP generation in normal liver cells (L02) when administered at doses up to 50 µM [61]. Other studies identified that oroxylin A increased activation of HNF-4α in HepG2 and SMMC-7721 cell lines. HNF-4α is a regulator in hepatocyte differentiation and a positive regulator of HIF-1α [62,179,180]. Oroxylin A also increases the PKM1/PKM2 ratio, leading to HNF-4α activation and subsequent differentiation of HepG2 cells and in SMMC-7721 cells at 8 and 20 µM concentrations, respectively [62]. Differentiation of these cells prevents them from proliferating uncontrollably and allows the transition into functional non-malignant cells of the liver.

Quercetin is a flavonoid found mainly in fruits and vegetables such as kales, berries, apples, red grapes, broccoli, cherries, radishes, onions, and in tea and red wine [64]. Quercetin shows regulatory effects on internal and external ROS-mediated protein kinase C signaling, preventing oxidative damage in the body through anti-oxidant activity, and inducing apoptosis in cancer cells [63,64]. In HepG2 cells, quercetin administration leads to upregulation of p53 and Bax [65,66]. Another study found that quercetin inhibited glycolysis in Bel-7402 and SMMC-7721 cells at a 50 µM dose by reducing levels of HK-2, which is a key enzyme involved in glycolysis that is overexpressed in HCC [67,181,182]. Further, glycolytic inhibition was also associated with a reduced expression of phosphorylated mTOR and Akt. Quercetin suppresses the AKT/mTOR pathways in SMMC-7721 and Bel-7402 cells in a dose-dependent manner (when tested at 12.5, 25, and 50 µM concentrations) by reducing HK-2 protein levels. Treatment with quercetin also inhibited the growth of HCC xenograft tumors in 5-week-old female nude mice when administered intraperitoneally at 50 mg/kg twice a day for 18 days. The ability of quercetin to inhibit the growth of the tumors was shown to be due to its capacity to reduce the levels of HK-2 expression [67].

A natural flavonoid, fisetin, is present in many fruits and vegetables, such as strawberries, apples, cucumbers, grapes, onions, and persimmons [183]. Fisetin has demonstrated its anti-oxidative, anti-metastatic, and apoptotic capabilities on different types of cancer, including HCC [184,185,186,187]. Fisetin dose-dependently reduced the cell viability and clonogenicity of HepG2 cells, arrested the cell cycle in the G2/M phase, and induced morphologic changes, when given at 25, 50, and 100 µM. Fisetin induced cell death via two mechanisms: apoptosis and necroptosis. An increased number of apoptotic cells were identified through DAPI staining and visualization of tightly condensed chromatin, indicative of cells entering an apoptotic state. Further, morphologic changes, including nuclei condensation, membrane blebbing, apoptotic bodies, and cell shrinkage confirmed the apoptotic cell death in the HCC cells. Apoptosis was also determined through Annexin V/PI staining, with its initiation occurring via increased expression of TNFα and decreased expression of NF-κB [68]. Fisetin increased the expression of TNFα and IKκB and decreased NF-κB, pNF-κB, and pIKκB expression. Fisetin also reduced the expression of Bcl2, while it increased the levels of Bax, caspase-3, and PARP in HepG2 cells [68]. Further, fisetin induced increased expression of RIPK1, pRIPK1, RIPK3, pRIPK3, and MLKL confirmed that cell death also occurred by necroptosis.

Glabridin is a type of flavonoid further classified as an isoflavone. This compound is extracted from the roots of the licorice plant and is used as both a sweetener and natural cosmetic. In addition to its more pedestrian uses, this compound also possesses promising anti-cancer characteristics. Glabridin was shown to inhibit both TGF-β and SMAD2 signaling pathways in HepG2 and Huh-7 cell lines (at a 20 µM dose). It was also proven to target SMAD2-3′ UTR, which impacted the expression of SMAD2 [69]. Additionally, when administered to normal liver cells (L02 at a dose of 10 and 20 µM) no effect on cell viability was observed [69]. Glabridin significantly decreased the invasive capacity of Huh-7 and SK-Hep-1 cells in a dose-dependent manner (10, 20, and 40 µM). This effect might also possibly be due to the down regulation of migration facilitatory proteins such as MMP-9 and MMP-1 due to an up-regulation of tissue inhibitor of MMPs. In vivo studies demonstrated the suppression of tumor formation with glabridin treatment in a xenograft model. SK-Hep-1 xenografted 6-week-old male BALB/c nude mice were found to have significantly reduced tumors volume after being treated with glabridin intraperitoneally at 10 mg/kg for 28 days, when compared to untreated xenografted control mice. The evidence from these studies suggests a possible future usage of glabridin as a chemopreventive agent for HCC metastasis [70].

Genistein, an isoflavonoid present in soybeans [71], is shown to have preventive effects on Bel 7402 cells. Genistein inhibited tumor cell growth and induced cell cycle arrest in the G0/G1 and G2/M phases when treated at a 10 µg/mL concentration [71]. Six-week-old male BALB/c nude mice, when xenografted with Bel 7402 cells, were found to have reduced invasion after intraperitoneal treatment with genistein (50 mg/kg once a day for 15 days) showing that genistein can inhibit metastasis in vivo [71]. In the Hep3B liver cancer cell line, genistein (127.6 µM) was proven to work in combination with gefitinib (9.8 µM), an EGFR inhibitor [72]. Additionally, in C57BL/6 mice who underwent chemical induction of HCC and who were genistein fed through chow (80 mg/kg/day for 5 months), there was a remarkable reduction in HCC incidence compared to mice who were only fed regular chow. Genistein has also been shown to significantly increase phospho-AMPK in total liver tissue extracts from the treated mice, and in Hep3B cells at 1 and 5 µM treatment concentrations. Further, it also promotes apoptosis and suppresses pro-inflammatory responses, combatting liver damage [188].

Luteolin and kaempferol are natural tetrahydroxyflavones found in many fruits, vegetables, and medicinal herbs. Although luteolin and kaempferol are reported as highly anti-oxidant molecules, they have been shown to have a cytotoxic effect on hepatocellular carcinoma at concentrations of 12 mM for luteolin and 20 mM for kaempferol, via generation of ROS and subsequent release of cytochrome-c from the mitochondria [73]. Another study on luteolin showed that it exhibited apro-apoptotic effect on HCC cell lines Huh7 and HepG2 (when administered at 10 µM). HCC xenograft studies further displayed luteolin’s anti-cancer abilities, when xenografted mice treated with 50 mg/kg/day of luteolin displayed a significantly decreased tumor size compared to control DMSO-treated mice [74].

*Fagopyrum tataricum* (L.) Gaertn (tartary buckwheat) is a traditional Chinese medicinal herb whose roots are rich in a flavonoid compound called tatariside F (TF). TF is selectively cytotoxic to hepatocellular carcinoma cells, as demonstrated in both in vitro and in vivo experiments. When H22 HCC cells were exposed to TF (at a concentration of 10 mg/kg), p53 and Bax were found to be dramatically upregulated, while Bcl-2 was found to be downregulated. This effect was enhanced when HCC cells were treated with both TF and current chemotherapy drug cyclophosphamide at 25 and 10 mg/kg respectively. When HCC cells were treated with this combination, upregulation of p53 and Bax was found to be significantly augmented versus cells treated with cyclophosphamide alone. HCC cells injected into ICR mice were allowed to form tumors and were then treated with TF. Significant inhibition of tumor weight and growth was observed in the mice treated with 10 mg/kg of TF compared to control mice. This inhibitory effect of TF was strengthened when administered alongside cyclophosphamide, at doses of 25 and 10 mg/kg. The inhibition of tumor growth by combining TF and cyclophosphamide was greater than in mice treated with cyclophosphamide alone at 10 mg/kg. These results suggest that TF may have potential, not only as a standalone cancer therapy, but also as a synergistic compound when used in combination with existing cancer drugs to increase their efficacy [75].

*Pulicaria jaubertii* is an edible and aromatic plant native to Europe, Asia, and Africa that has been shown to repress cancer development in human lung cells and human hepatocellular carcinoma cells, even when compared to sorafenib (the current first-line drug treatment for HCC) [76]. Out of four different glycosides isolated from this plant, only one, *Pulicaria jaubertii 1* (PJ-1), has shown a great potential to inhibit mutated K-Ras/B-Raf protein expression through competitive inhibition. Importantly, this inhibition was found to occur only in HepG2 cancer cells and not in normal hepatocyte cells. No cytotoxicity was observed when normal hepatocytes were treated with doses up to 1.5 mg/mL. However, the same dose was effective in inducing cytotoxicity in HepG2 cells. Further, PJ-1 activated p53 protein while also decreasing TGF-beta and IL-8 expression levels [76].

Jujube leaf green tea extracts (JLGTE) also have shown potential as cancer therapeutics. Although the major bioactive compounds in JLGTE have yet to be elucidated [189], there is evidence that the major bioactive components in jujube leaves are flavonoids [189]. Nonetheless, JLGTE has shown to have anti-proliferative effects in HepG2 cells at 10 µg/mL, while not affecting normal hepatocyte LO2 cell line, even when treated at the same dosage of 10 µg/mL. JLGTE has also been shown to exert pro-apoptotic effects by activating AMP-activated protein kinase (AMPK) [77].

Icaritin is isolated from the plant *Epimedii herba*, traditionally used in Chinese herbal remedies [117]. Remedies involving icaritin have been long utilized, as it seems to have a role in immune modulation. Icaritin treatments have been shown to modulate natural killer (NK) cells, T cells, and myeloid-derived suppressor cells (MDSCs) [190]. Studies have demonstrated anti-proliferative activities of icaritin in cancer cells as well as cancer-stem cells [191]. However, there was little inhibitory activity noted when icaritin was administered to a normal liver cell line (L02 at doses up to 20 µM). In a clinical trial, icaritin showed a high tolerability with no immune-related adverse events in patients with advanced HCC when given at a dose of 600 mg orally twice a day as well as in the dose escalation to 800 mg [117]. Icaritin continues to show great promise for future HCC treatments as it is currently being evaluated in two Phase III trials [192].

#### Catechins

Green tea leaves (*C. sinensis*) also contain many compounds, which may have potential as cancer treatments. Although extract from green tea leaves contains dozens of organic compounds (including caffeine and methylxanthines), the polyphenols found in green tea extract have been most coveted for their antioxidant nature and numerous health benefits. Additionally, in green teas, all these compounds stay relatively well preserved, increasing their potency and efficacy.

Epigallocatechin-3-gallate (EGCG) is one of the polyphenols extracted from green tea that displays promising anti-cancer characteristics. It has been shown that EGCG may impede HCC proliferation in Hep3B, HepG2, SK-Hep1, HCC-LM3, Huh-7, and SMMC-7721 cell lines by inhibiting the ERα36, which is the primary estrogen receptor on HCC cells [78]. EGCG significantly inhibited the proliferation and enhanced the pro-apoptotic effects in HCC cells expressing high levels of ERα36 when treated with 30 or 60 µg/mL concentrations. Further, Hep3B cells with high ERα36 expression showed a dose-dependent inhibitory downstream effect due to EGCG exposure. The downstream effects were found to be both anti-proliferative (via the inhibition of PI3K/Akt and MAPK/ERK pathways) and pro-apoptotic (via the ERα36-EGFR-Her-2 feedback loop and caspase 3 activation) [78]. Numerous other studies support the ability of EGCG to inhibit HCC growth and metastasis. It has been shown that EGCG inhibits HIF1 α protein accumulation in HepG2 cells treated at 80 µg/mL by altering the PI3K/Akt and ERK1/2 signaling pathways [79,80]. Furthermore, it was reported that EGCG induced apoptosis in HCC cells by decreasing the mitochondrial membrane potential and impeding proliferation through induction of G0/G1 cell-cycle arrest [79,81]. EGCG was also shown to induce apoptosis in Hep3B cells through the activation of caspase-9 and caspase-3 when treated at a dose of 10 µg/mL [79,82]. Previously, a 186-gene signature was found in cirrhotic liver tissue to predict the risk of HCC [193]. Using these parameters, a study that used diethylnitrosamine (DEN), a hepatocarcinogen, to induce HCC in rats found the 186 gene “poor prognosis” signature to be absent from the gene expression profile of rats treated with EGCG [83]. This is a strong indicator that EGCG can attenuate the development of HCC. Additional in vivo studies may be warranted to support the anti-cancer effects of EGCG. When DEN-induced HCC male Sprague Dawley rats were intragastrically treated with EGCG (at a dose of 25 mg/kg) their tumor volume was significantly decreased, and their survival rates were increased [84]. *Camellia sinensis* (L.) O. Kuntze cv. CFT-1 is a tea variety, which was specifically bred to be high in EGCG. When male Wistar rats with HCC were exposed to tea infusions of CFT-1 via incorporation into drinking water for 20 weeks, hepatic nodules were significantly reduced in size, number, and incidence compared to non-CFT-1 treated rats [85]. EGCG has also been found to change the transcriptome of HCC cells. When Hep3B HCC cells were exposed to stimuli that mimicked the tumor microenvironment and then treated with EGCG, 922 genes expressions were found to have been altered when compared to cells that were not exposed to the tumor microenvironment mimicry [86]. miR483-3p expression correlated with decreased survival and increased HCC progression. In an HCC mouse model, EGCG treatment via incorporation of 0.1% and 0.5% EGCG into drinking water was found to reverse the mir483-3p-induced enhancement of HCC cell migration and invasion. Additionally, EGCG was able to impede mir483-3p-induced modification of the expression of EMT markers: E-cadherin and vimentin. EGCG also downregulated the endogenous expression of mir483-3p in HCC cells [87]. EGCG has also shown promise when used in conjunction with other treatments. Typically, in many cancer cases, activation of prostaglandin receptor EP1 induces cell migration and invasion, leading to the occurrence of metastasis. EGCG, when combined with EP1-selective antagonist ONO-8711, effectively inhibited HepG2 cell viability and migration ability at an increased rate than EGCG alone (when treated at doses of 12.5, 25, 50 and 100 µg/mL) [88]. These findings shed light on the specific mechanisms by which EGCG suppresses HCC growth.

#### 3.2.2. Stilbenes

Pterostilbene, a naturally found stilbenoid related to resveratrol as a methoxylated analogue, is commonly found in blueberries, several types of grapes, and tree wood. It is considerably more bioavailable due to its two methoxy groups compared to resveratrol, leading to it becoming a compound of interest for cancer treatment [194]. Pterostilbene has an inhibitory effect on almost every cellular process that leads to metastasis in an apoptosis-dependent and apoptosis-independent manner. It exhibits an anti-metastatic ability by preventing tumor cell colonization and decreasing secondary tumors in distant organs, which was found when pancreatic cancer nude mice model were treated with 100 µg/kg/day and 500 µg/kg/day of pterostilbene [89]. Inhibition of HCC growth was shown when SMMC-7721 cancer cell line was treated with 50 µM of pterostilbene. Inhibition seem to be achieved via the downregulation of the Metastasis-Associated Protein 1 (MTA1) and histone deacetylase 1 (HDC1) complex. Destabilization of this complex increases the activation levels of the tumor suppressor PTEN in tumors, which ultimately promotes apoptosis in HCC [90]. Future endeavors can turn pterostilbene into complementary medication to reduce the rate of metastasis in HCC.

Resveratrol is a natural product commonly found in grapes, peanuts, and pines with known anti-oxidative, anti-inflammatory, and cytoprotective effects [91]. Resveratrol oligomers have been shown to be cytotoxic to HepG2 cells at 21 µM through induction of cell cycle arrest at G2/M, increased intracellular ROS, and increased caspase-3 activity [92]. Additionally, resveratrol has been shown to inhibit HCC proliferation, migration, and invasion through downregulation of membrane-associated ring-CH–type finger 1 (MARCH1), an E3 ubiquitin ligase typically found to be heavily upregulated in HCC [93]. Resveratrol treatment, when administered orally to rats with HCC at a dose of 100 mg/kg for 2 weeks, restored the antioxidant enzymes catalase and glutathione peroxidase to normal levels, as these are typically downregulated in HCC [94]. A resveratrol diet fed to Sprague Dawley rats (at a dose of 300 mg/kg) was shown to reduce nitric oxide production in the liver through inducible nitric oxide synthase downregulation [95]. Hexokinase 2 (HK2) is an enzyme known to change the metabolic phenotype of cells to support anaerobic growth. Resveratrol administered to HCC-LM3 cells at a concentration of 20 µM inhibited HK2 expression [96]. Combined with sorafenib, resveratrol inhibits HCC cell growth and increases the anticancer effect of the drug through increased suppression of anaerobic growth [96]. In DEN-induced HCC rat models, resveratrol was found to be an effective cancer therapeutic indicated by a significant increase in apoptosis [97]. When resveratrol was administered to an HCC mouse model, it was found that tumor growth was inhibited through a marked decrease in the frequency of CD8+CD122+Tregs in the tumors, lymph nodes, and the spleen of the mouse. IFN-γ expressing CD8+ T cells in the tumors and peripheral lymphoid organs were also found to be increased [32]. The effects of resveratrol seem to be enhanced when the compound is able to be more precisely delivered to the cancer site. Further, the anticancer effects of resveratrol were improved when delivered precisely to HCC via nanoparticle delivery system than when traditionally administered [98]. Another study also found similar success with a nano delivery system using cationic liposomes to deliver resveratrol; it significantly reduced the number of liver nodules [99].

#### 3.2.3. Xanthones

*Garcinia mangostana,* also known as the mangosteen, is a popular tropical fruit grown in Southeast Asian countries known for its sweet taste. Two unique compounds isolated from the *G. mangostana* were reported to have anti-cancer effects. They are two xanthones: mangostanaxanthone V and mangostanaxanthone VI [100]. Both xanthones were found to inhibit the proliferative potential of HepG2 cells significantly. Furthermore, mangostanaxanthone V halted the cells in the G0/G1 phase of the cell cycle, while mangostanaxanthone VI was found to have an impact on G2/M phases [100].

Alpha-mangostin is another active xanthone compound in *Garcinia mangostana*. When administered at a dose of 5.5 µM, it sensitized HepG2 cells to anoikis [101], a type of programmed cell death that occurs when anchorage-dependent cells detach from their extracellular matrix. This process is vital to the metastatic spread of malignancies [195]. It was found that alpha-mangostin greatly sensitized HepG2 cells to anoikis through the stimulation of pro-apoptotic mechanisms such as induction of caspase-9, caspase-8, and caspase-3, and decreased anti-apoptotic protein levels. Alpha-mangostin significantly reduced the re-adhesion and migration of HepG2 cells (when administered at a dose of 2 µM) through the inhibition of MMP-2 and MMP-9, along with suppression of molecules involved in AKT and ERK signaling pathways. These findings suggest that alpha-mangostin can potentially be an effective multi-target drug to treat HCC [101]. Interestingly, the various doses of alpha-mangostin (10, 20, 30, 40, and 50 µM) tested on normal human osteogenic cells showed no change in cell viability, which indicates that alpha-mangostin had only minimal cytotoxicity to normal cells [196].

Gambogic acid (GA) is a xanthone isolated from gamboge, a dry resin from the trees of the genus *Garcinia*. Traditionally, gamboge has been used as an anti-inflammatory, parasiticide, and detoxification medicine for thousands of years throughout Southeast Asia [197]. Gambogic acid was shown to have an anti-cancer effect on SMMC-7721 cells through necrosis and reduced proliferation when administered at a concentration of 0.625–5.0 mg/mL, and increased inhibitory effect on telomerase when administered at a dose of 2 mg/mL [104]. In another study, GA induced apoptosis through oxidative stress in HepG2 cells when administered at a concentration of 0.75–12 µM [105]. Malignant cells overexpress thioredoxin reductase (TrxR) isoenzymes for their tumor growth. Functional TrxR also plays an important role in decreasing the oxidative stress load in cells. GA inhibits TrxR1 activity to induce oxidative stress and then apoptosis in SMMC-7721 HCC cells when given at a dose of 3 µM [106].

### 3.3. Alkaloids

Alkaloids are nitrogen-containing compounds found in abundance in many plants. Alkaloids have been used for therapeutic purposes for many years and are well known for their healing and medicinal properties [198]. Alkaloids (such as caffeine) can be found in abundance in coffee, the consumption of which is associated with a lowered risk of HCC, as well as with a lowered risk of chronic liver disease. A population-based prospective cohort study of more than 215,000 men and women found that regardless of ethnicity, sex, BMI, smoking status, alcohol intake, or diabetes status, participants who consumed coffee had a comparatively lower incidence of hepatocellular carcinoma and chronic liver disease [199]. In an 18-year follow up period study, when compared to non-coffee drinkers, those who consumed two to three cups per day showed 38% reduction in risk for developing HCC. Those who drank four or more cups a day during the same follow up period were found to have a 41% reduction in HCC risk [199]. In a meta-analysis, people who drank coffee were less likely to develop cirrhosis or fibrosis of the liver compared to non-coffee drinkers. [200]. Fibrosis and cirrhosis increase the risk of HCC; therefore, pathways that lead to development of these diseases could be potential targets for HCC treatment. The antioxidant effects of coffee in the liver are associated with Nrf-2, a transcription factor that binds to anti-oxidant response element (ARE) to promote many anti-oxidative genes. Proteins and enzymes known to be regulated by Nrf-2 showed an increase in expression as the concentration of coffee increased, resulting in an overall reduction of oxidative stress [201]. Interestingly, there was not found to be much of an HCC chemo preventative effect in people who drank decaffeinated coffee compared to non-coffee drinkers, suggesting caffeine may play a large role in coffee’s chemo preventative ability [202,203]. Additionally, more specific studies concerning the treatment of HCC have shown that combination therapy of caffeine with 5-fluorouracil, compared to therapy with only 5-fluorouracil, demonstrated decreased cellular proliferation and a significant increase in apoptosis via regulation of intracellular ROS production [41].

*Rhizoma coptidis,* also known as the Chinese goldthread, is a flowering plant native to China whose roots have been used widely in China for thousands of years to treat many different ailments and diseases [204]. Recently, evidence has emerged suggesting the herb may have potential as a future therapeutic option for the treatment of HCC. Berberine, (an isoquinoline derived alkaloid), is the major compound found in *Rhizoma coptidis*. Berberine causes apoptosis and has dose-dependent (10, 50, and 100 µM) anti-proliferative effects on HepG2 cells [102]. Additionally, 50 µM berberine-treated HepG2 cells had reduced levels of NF-κB-p65, suggesting berberine may be acting through p65 to suppress the NF-κB pathway [102].

Neferine is a natural bisbenzylisoquinoline alkaloid derived from the green seed embryos of the flowering aquatic plant, Indian Lotus (*Nelumbo nucifera*). Neferine induced apoptosis in Hep3B cells at doses over 10 µM by downregulation of proliferative genes c-Myc, cyclin D1, D3, CDK4, and E2F-1 and inducing ER stress [103]. Yoon et al., demonstrated that neferine also exhibited selective cytotoxicity and apoptosis towards HCC cells and not in THLE-3 normal liver cells. With the normal liver cells, there was minimal loss of cell viability even after treatment with 30 µM of neferine.

The plant alkaloid irinotecan originates from the *Camptotheca acuminata* plant. Irinotecan is a topoisomerase-1 inhibitor which interferes with DNA synthesis by causing single stranded DNA breaks and inhibiting DNA repair enzymes [205]. Therapeutic potential of the irinotecan compound has been demonstrated and it is FDA approved for metastatic colorectal cancer [205]. In a clinical study, patients with HCC were treated with irinotecan at a dose of 125 mg/m^2^. Although patients had a modest result of improvement after treatment, toxicity with this compound is a real concern.

### 3.4. Polysaccharides

Polysaccharides are long chains composed of simple sugars. They are imperative to life, and are found in a wide variety of plants, animals, and fungi.

Active hexose correlated compound (AHCC) is a *Basidiomycotina* polysaccharide extract derived from the hybridization of mushrooms [206]. AHCC has been shown to play a role in immune cell function and cell number; such immune cells include natural killer (NK) cells and T cells, which can possibly defend the host from infections and malignancies [207]. When administered with a high dose of 9 g of liquid AHCC per day, no significant abnormalities in laboratory parameters in healthy subjects was reported [208]. Overcoming high toxicity levels from most compounds brought into a clinical setting is always an obstacle, but, as stated in the previous study, AHCC has a potentially low toxicity in humans, therefore, making a viable option as a therapeutic agent. The addition of AHCC at 360 mg·kg^−1^·d^−1^ with a low dose of 5-fluorouracil chemotherapy at 10 mg·kg^−1^·d^−1^ treatment in mice resulted in a greater antitumor effect and caused more apoptosis in tumor tissue [209].

### 3.5. Whole Extracts

Whole extracts derived from plants may contain more than one phytochemical, and they hold much promise as future cancer therapies. *Poncirus fructus* (PF) is a methanol extract from the immature dry fruit of *Poncirus trifoliata*. This deciduous shrub grows naturally in the South Korean islands of Jeju and Gaduk, and East Asian cultures have long used its fruit to treat disorders ranging from gastrointestinal to cardiovascular [107]. In addition to being beneficial for the aforementioned health issues, PF also seems to contain promising anti-cancer properties. PF has demonstrated cytotoxicity against HCC cells, inducing apoptosis and significantly inhibiting the proliferative and colony-forming abilities of Hep3B and Huh7 in a dose-dependent manner when treated at 20, 30, and 40 µM doses in vitro [107]. PF seems to induce cytotoxicity in HCC cells through mitochondrial-mediated apoptosis pathway; PF induced the loss of mitochondrial membrane potential, which in turn caused high levels of intracellular ROS generation. Additionally, PF inhibited the migratory and invasive capabilities of HCC cells by increasing the expression of epithelial marker E-cadherin while simultaneously downregulating mesenchymal and cell cycle markers B-catenin, N-cadherin, vimentin, Snail, a-SMA, Pin1, and cyclin D1 [107]. Furthermore, PF downregulated MMP-2 and MMP-9. These MMPs are crucial players in the ability of tumor cells to invade, as they assist in basement membrane degradation. Such attenuation of MMP-2 and MMP-9 activity suggests PF may be a useful compound in preventing the spread of HCC.

*Trametes robiniophila,* also known as Huaier, is a mushroom that has been used as a traditional Chinese medicine for over 1000 years [210]. The most common preparations of Huaier include granules or aqueous extracts. The main active ingredient, proteoglycan, consists of amino acids, polysaccharides, and water [211]. Huaier has potent anticancer effects on HCC [212], breast cancer [213], and more. When administered to HepG2 HCC cells at a dose of 15 mg/mL, the granules of this mushroom decreased proliferation and migration [214]. Additionally, Huaier granule did not cause cytotoxicity in L-02 normal human hepatocyte cells, indicating it is targeting only HCC cells [214]. In one randomized controlled clinical trial, ingestion of an aqueous extract from *Trametes robiniophila* (20 g in 100 mL water, 3 times a day for 96 weeks) significantly lowered cancer recurrence and metastasis rates, while also improving survival rates in patients who underwent liver resection for HCC [215].

*Smallanthus sonchifolius leaf (YLE*) or Yacón is a species of perennial daisy, which has traditionally been grown in the northern and central Andes from Colombia to Northern Argentina for its sweet tasting roots. In the early 2000s, YLE was cultivated in Myanmar, and since then has been used as a traditional herbal remedy in rural parts of Myanmar to treat liver diseases. Recently, it has been demonstrated that whole plant methanolic YLE extract possesses significant anticancer activity against HepG2 cells (with an IC_50_ of 58.2 µg/mL). When administered to HepG2 cells, YLE significantly induced necrosis without activating caspases 3 or 8, and induced cell cycle arrest in the cell line. YLE also decreased ROS in both dose and time-dependent manners, lending support to its inhibitory effects on cancer cell proliferation [216]. Interestingly, cell viability of HEK 293 non-tumor cells was not affected by YLE (0–100 µg/mL), indicating cytotoxicity is specific to HCC cells [216].

*Cyperus amuricus,* also known as Asian flatsedge, is a grass native to China, Japan, Korea, and European Russia that has been used in oriental folk medicine as an astringent, for wound healing, as a diuretic, and for other intestinal problems [217]. Whole plant methanolic extract of *C. amuricus* demonstrated cytotoxic effects against Hep3B cells at 100–200 µg/mL, and suggested that the cell-death induced by the *C. amuricus* extract was through an apoptotic mechanism, based on the morphological changes associated with the 24-h treatment. Treatment of Hep3B cells resulted in inhibition of CDK’s and cyclins, leading to cell cycle arrest at sub-G1. Data also suggests that FADD cleavage of procaspase-8 into activated caspase-8 may be triggered by *C. amuricus*. Then, the subsequent caspase-8 regulated cleavage of Bid into tBid and tBid’s translocation to the mitochondria may enable the crosstalk between the intrinsic and extrinsic apoptotic pathways. Furthermore, the ordered activation of cleaved caspases-8, -9, -3, -7, -6, and cleaved PARP along with other proteins related to apoptosis lend further support to the idea that *C. amuricus* anticancer properties are related to its ability to induce apoptosis [217]. Importantly, *C. amuricus* (50–200 µg/mL) did not have significant cytotoxic effects on non-cancerous human keratinocytes [217].

Fraxini-2 comes from the *Viscum album* L. mistletoe plant, derived from ash trees, which contains many biologically active substances [114]. Fraxini-2 has been shown to have natural killer cell activity as a result of upregulated production of interferon gamma (INF-γ), tumor necrosis factor-α (TNF-α), activation of monocyte-macrophages and helper cells, and higher levels of cytokines [114]. In a clinical study, 12 patients with advanced HCC were given fraxini-2 by subcutaneous injection (at a dose of 20 mg once per week for two weeks) [218]. Patients were on this treatment regimen for varying times, ranging from 2.6 to 36.4 weeks. Generally, alpha-fetoprotein (AFP) levels of patients seemed to stabilize within the first 3–4 weeks of fraxini-2 treatment. High AFP levels are historically used as serum markers for the presence of HCC [219], as such, lowered AFP levels observed after fraxini-2 treatment are encouraging results. The evidence from this study seems to indicate that there may be a place for AFP as a supplemental treatment option in the future; however, there needs to be more studies done. The total population of this study was very small, with results for only 10 people being available at the end. Additionally, AFP may only prove beneficial to specific HCC patients, so future studies controlling for previous treatments and disease characteristics would be required in order to be able to draw convincing conclusions.

### 3.6. Combination

Phytochemicals may gain efficacy due to their synergistic anti-cancer effects when combined with other compounds or phytochemicals. The potential that these combinations may have as future anti-cancer therapies is promising and warrants further exploration.

Moreover, 1′-Acetoxychavicol acetate (ACA) is an acetate ester derived from the rhizomes of *Alpinia galangal* of the ginger family. It is commonly used as a traditional medicine, and as a spice in the culinary arts. ACA induced apoptosis in HepG2 cells when administered at 10^−5^ M [220]. ACA has been shown to be beneficial in preventing the advancement of carcinogenesis on chemically-induced tumors, when administered to rat intestinal epithelial cells [221]. ACA was also found to be a beneficial anti-cancer compound when used together with sodium butyrate. Sodium butyrate is a short-chain fatty acid mainly produced by bacterial fermentation of dietary fibers that has previously been shown to have anti-cancer effects [222,223,224,225]. The combination treatment of ACA and sodium butyrate caused a significant increase in reactive oxygen species and NADPH oxidase activities in HepG2 cells [108]. This combination treatment also showed a synergistic inhibitory effect on HCC growth, which was strongest when 12.5 µM of ACA was combined with 2 mM sodium butyrate. Furthermore, it significantly increased the levels of AMPK. The ACA plus sodium butyrate combination treatment could be used as targeted therapy for HCC without causing significant liver toxicity [108]. The combination index was found to be less than 1, further indicating a strong synergistic relationship between ACA and sodium butyrate.

Betulinic acid (Bet A) and ginsenoside Rh2 (G-Rh2) have been shown to induce apoptosis in HepG2 cells. Bet A, a pentacyclic triterpenoid, is derived from white birch trees. Individually, it promotes pro-apoptotic cell death in various cell types through the mitochondrial pathway [226,227,228]. G-Rh2 is isolated from the root of *Panax ginseng* [229,230]. Ginseng has been traditionally used in herbal medicine in eastern Asia, with its major active ingredient being ginsenosides. Ginsenosides have exhibited effects on immune response, metabolism, and cancer treatment and prevention [231,232]. G-Rh2 alone moderately induced apoptosis in HepG2 cells; however, in conjunction with Bet A, it had more pronounced effects on HCC proliferation and apoptosis, strongest at doses of 10 µg/mL of Bet A combined with 12.5 µg/mL of G-Rh2. Such processes were evidenced by morphologic changes characteristic of apoptotic cells, including nuclear fragmentation, membrane blebbing, and nuclear condensation [109]. Combination index of these compounds was less than 1, indicating a synergistic effect.

Huanglian decoction is comprised of four traditional Chinese herbal medicines: *Coptidis Rhizoma*, *Zingiberis Rhizoma*, *Folium Artemisiae Argyi*, and *Mume Fructus*. The active compound of this mix is the alkaloid berberine hydrochloride (0.26 mg/mL). Huanglian decoction has a direct negative effect on the growth of liver cancer cell lines. Their ability for migration and invasion is impeded through induction of apoptosis and G2/M phase cell cycle arrest. Additionally, downregulation of G2/mitotic-specific cyclin-B1 (CCNB1) activates the p53 pathway through the upregulation of Bax, caspase-3, caspase-9, p21, and p53 [110]. Huanglian decoction further shows its remarkable effects in vivo [111], whereby tumor growth and angiogenesis in a xenografted murine model was found to be suppressed after treatment with the decoction. The mechanism of action is hypothesized to be through attenuation of eEF-2 [112], which is an enzyme found to be upregulated in various cancers [233].

Traditional Chinese medicine (TCM) also include compounds, such as Fufang Banmao Capsule, and Huaier Granule [234]. Fufang Banmao has a long history of being used to treat HCC. In one study, 320 patients with HCC were given Fufang Banmao in a 0.25 g/capsule form, to be taken orally twice daily for 6 months. When compared to the control group in the study who did not take the capsule, patients taking Fufang Banmao showed a significant increase in overall survival rates [235]. Huaier Granule is specifically derived from a mixture of fermentation products that originate from the plant *Trametes robiniophila Murr* [234]. This granule has also shown evidence of having anticancer effects against HCC, most notably through promoting apoptosis through p38-MAPK, inhibiting metastasis and angiogenesis, and also causing cell cycle arrest in the G0/G1 phase [234]. In a clinical trial of 340 people, Huaier granule was found to prolong progression-free survival of HCC patients who had previously undergone thermal ablation. Those patients who were taking the Huaier granule for more than 2 years through oral administration saw the most benefit [116]. These compounds have also been shown to reduce HCC risk in patients with Hepatitis B and reduce risk of cirrhosis [236,237]. Studies have shown the potential of such phytochemicals to inhibit HCC cell growth, induce cell death, and reduce the inflammatory response, as well as increase antiviral activity.

Jianpi Huayu therapy is another TCM that consists of ginseng, atractylodes, tuckahoe, licorice root, *radix bupleuri*, yam, *cortex moutan*, *salvia miltiorrhiza*, turmeric, and *rhizoma zedoariae* [120]. TCM has shown to be abundant in therapeutic effects including reducing radiation/chemotherapy toxicity, improving patient quality of life, and prolonging patient survival [234,238,239] and Jianpi Huayu seems to be no exception. Jianpi Huayu therapy has been reported to be beneficial to HCC patients in a clinical trial when administered post-hepatectomy in a clinical trial [120]. Additionally, one study attempted to explore the underlying mechanism of Jianpi Huayu therapy and its effectiveness against HCC. SMMC-7721 cells were orthotopically implanted into 4-to-7-week-old nude mice. Once tumors were present, the mice received 24.96 g/kg of the Jianpi Huayu compound intragastrically for 21 days, while the control mice received saline. By the end of the experiment, mice with Jianpi Huayu treatment had significantly reduced tumor size and weight compared to the saline-fed control mice. The study speculates that HCC suppression may be due to Jianpi Huayu’s ability to downregulate miR-602 levels, which are typically upregulated in patients with liver cancers [240]. Although this study had promising results, the use of such a high dose of Jianpi Huayu is a little concerning when it comes to translatability to human beings. Further research is necessary to determine the toxicity of this compound in humans, in order to establish a safe maximum dosage level. However, it is also important to note that there were no instances of toxicity in the clinical trial [120].

The Jianpi Ligan decoction is made up of several TCM components involving *Radix Codonopsis*, *Rhizoma Atractylodis macrocephala*, *Poria cocos*, *Radix Glycyrrhizae*, *Rhizoma Diosscoreae*, *Rhizoma Pinelliae*, *Fructus Crataegi*, *Semen Nelumbinis*, *Herba Artemisia scoparia*, and *Pericarpium Arecae* [121]. In two retrospective clinical trials, Jianpi Ligan decoction, when given in conjunction with either transcatheter arterial chemoembolization (TACE) or radiofrequency ablation (RFA), showed a significantly improved overall survival compared to patients who received only TACE or RFA treatments [121,122].

Shenqi mixture is a type of TCM blend of many herbs consisting of pseudostarwort root, milkvetch root, poria, white atractylodes tuber, rehmannia root, dendrobium, white peony root, Chinese angelica root, oldenlandia herb, Chinese lobelia, wild ginger, and licorice root [129]. Shenqi mixture medicine has benefits such as improving blood circulation and resolving toxins [129]. This combination of herbs was shown to be successful in a clinical trial where 20 mL (containing 20 g of the crude mixture) was orally administered to patients (who had previously received microwave coagulation) three times a day. Effectivity rate of the treatment in treatment group was 75.00% compared to the control group.

## 4. Conclusions

Hepatocellular carcinoma is a rapidly rising deadly disease that has few therapeutic options currently available to patients. HCC becomes even more lethal once metastasis occurs, and, to make matters worse, the treatment options at this stage dwindle even further. There is a high demand to explore untapped natural reservoirs in order to produce medications and treatment options for patients who far too often have none. Fortunately, many phytochemical compounds show promise as therapeutic options for patients with HCC metastasis. Additionally, phytochemicals also have a great potential as adjuvant therapies. Several studies have shown the safety, efficacy, feasibility, and mechanism of actions of phytochemicals supporting their incorporation into current chemotherapies.

However, it is important to consider the shortcomings and limitations that come along with many of these investigative processes. Often, in vitro studies may prove to be more misleading than enlightening. Cell culture experiments cannot possibly replicate the complexities of a whole biological organism, and, for this reason, the possibility exists that though a compound is seen to have one effect in vitro, there may be entirely another effect noted in in vivo models. However, in the worst cases, there may actually be a severe toxicity in the organism, even if no such toxicity was noted in cell lines. In regard to in vivo testing, it is important to keep in mind that many of this research uses rodents as biological models to explore the potential effects of new compounds, because they are easy to handle and accessible. This itself has its limitations, as rodents have different metabolisms and characteristics from a human being. Once again, even though a compound shows promise in vitro and in vivo, it may not be able to be translated effectively over to humans.

There is also the obstacle of statistical power and population size. For many of the clinical trials discussed here, the number of patients tested was quite small. Smaller populations limit the power of the clinical trial, as the results (even if very positive) cannot safely be applied to a large population based off the results and side effects experienced by only a small number of humans. Small clinical trials are themselves foundations for larger clinical trials, whereby more people being involved can truly help scientists and physicians come to a more statistically powerful conclusion.

Dosing becomes a serious issue as well when considering the translatability of many of these compounds. Although there may be positive effects seen in pre-clinical studies, the effects may possibly be due to high doses being distributed onto cells or animals, which would not be translatable over to humans. Once again, toxicology becomes a real concern when trying to bring over new treatments and medications to the clinics from pre-clinical studies. It would be best to use the lowest dose with the maximum benefits, but the effective doses used in various studies differ drastically. It is well known that no phytochemicals are equally active. The efficacy and potency of the phytochemicals depends on several different parameters like drug bioavailability, absorption, clearance, etc. Some of them might be required in large quantities to produce a beneficial effect, while others might be needed only in very minute quantities. Some phytochemicals are effective at high doses and that could be due to the fact that the active ingredient might be in low concentrations. Therefore, the effective doses are expected to vary from phytochemical to phytochemical.

Further, there is a lack of standardization techniques when it comes to these compounds. There are, for example, differing methods of extraction, maintenance, storage, and administration of these phytochemicals. All of these variations may have a profound effect on the activity of the compounds, and may account for why one research lab may see significant results and another may not. Until there is a more standardized practice of extraction and handling for each compound, results may be highly variable and, therefore, unreliable. Additionally, due to lack of standardization, some compounds may inadvertently contain toxins, which could skew results through causing harm that is not a result of the compound itself. It is paramount that caution be taken about the quality of phytochemical compound that is being tested. For these reasons, there remain many obstacles between foundational experimentation and actual clinical application.

Regardless, the scientific processes we employ to make new discoveries is often enough effective, as evidenced by the daily advancements in science and medicine using the same approaches as discussed in the studies reviewed here. Although a great deal more information is needed for many of these phytochemicals before they can become full-fledged clinical treatments, the foundation for this branch of medicine seems very strong.

**Figure 3:**
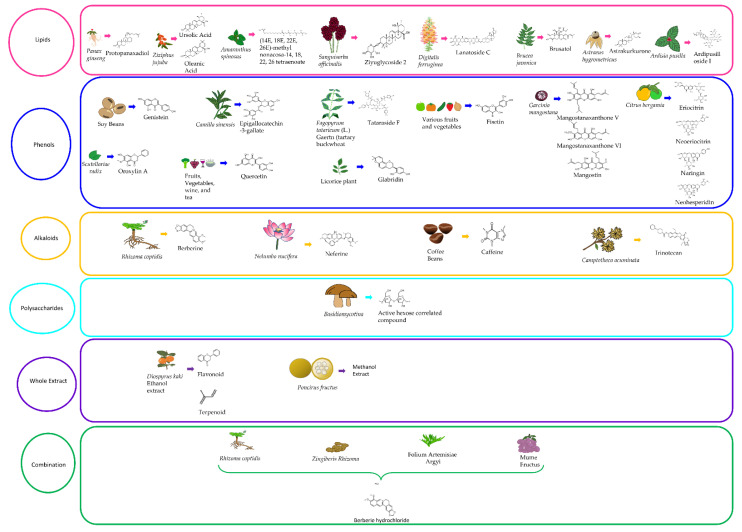
Phytochemical compounds against HCC fall into several categories: lipids, polyphenols, alkaloids, whole extracts, and phytochemical combinations.

In this review, we have discussed the effects phytochemicals on HCC from preclinical and clinical studies and have found encouraging and promising outcomes. The phytochemicals reported in this review were classified into several categories such as lipids, polyphenols, alkaloids, polysaccharides, whole extracts, and phytochemical combinations (Figure 3). We strongly advocate that more scientific attention is needed on phytochemicals to continue to assess their beneficial effects and combat HCC. Of particular importance is the need to expand upon the areas of bioavailability, pharmacokinetics, and pharmacodynamics of many of these compounds. More investigation into how phytochemicals can be used as effective anticancer drugs may be the key to bridging the gap between preclinical studies and clinical studies for many of these phytochemicals. Almost 80% of the compounds failed to progress into clinical studies due to lack of information regarding efficacy, bioavailability and unanticipated toxicity to normal cells [241]. Although there remain large obstacles, phytochemicals can be used either as an alternative or integrative therapy in conjunction with existing HCC chemotherapies. The use of predictive non/pre-clinical models and organ-on-a chip technology that resemble human physiology more precisely is crucial to assess the pharmacokinetics, pharmacodynamics, efficacy, and safety of therapeutic phytochemicals. In conclusion, phytochemicals have great potential as treatment options for hepatocellular carcinoma.

## Figures and Tables

**Table 1:** List of natural compounds and their mechanism of action on hepatocellular carcinoma from preclinical studies.

Plant	Compound	Study Type	Cell Type	Mechanism of Action	References
Amaranthus spinosus	(14E, 18E, 22E, 26E)-methyl nonacosa-14, 18, 22, 26 tetraenoate	In vitro	HepG2	Inhibition of proliferation by upregulation of Bax and downregulation of Bcl-2 and cyclin B resulting G2/M arrest.	[46]
Astraeus hygrometricus	Astrakurkurone	In vitro	HepG2, Hep3B	Inhibition of proliferation through cycle arrest at sub-G0/G1 phase, upregulation of pro-apoptotic markers Bax and cleaved caspase 9, with downregulation of antiapoptotic marker Bcl-2.	[47]
Ziziphus jujuba	Ursolic acid	In vitro	HepG2, Huh-7	Inhibition of proliferation through disruption of DNA fork establishment, causing cell cycle arrest. Increased expression of p21/WAF1, inducing cell cycle arrest and apoptosis. Inhibition of expression of XIAP mRNA, typically elevated in cancer cells.	[48,49]
	Oleanolic acid	In vitro	Huh-7	Induction of apoptosis through increased mitochondria permeability, leading to activation of pro-apoptotic markers. Inhibition of expression of XIAP mRNA, typically elevated in cancer cells.	[50]
Panax Ginseng	Protopanaxadiol (PPD)	In vitro	HepG2, PLC/PRF/5	Inhibition of EMT through increased expression of E-cadherin and decreased expression of vimentin. Inhibition of EMT also through prevention of STAT3 activation and through inhibition of Twist1 expression.	[51]
Ardisia pusilla A. DC.	Ardipusilloside I	In vitro In vivo	HepG2, SMMC-7721	Inhibition of survival, invasion, and metastasis through suppression of MEK/ERK and Akt signaling pathways. Additionally, impedes metastasis through upregulation of E-cadherin.	[52]
Digitalis ferruginea	Lanatoside C	In vitro	Huh7, PTEN deficient Mahlavu human liver cancer cells	Inhibition of proliferation through cell cycle arrest in G2/M. Induction of apoptosis through JNK pathway activation, and ROS production.	[53]
Diospyros kaki	Non-specified terpenoids and flavonoids	In vitro	HepG2, Hep3B, SNU475	Inhibition of epithelial to mesenchymal transition through inhibiting expression of snail, vimentin, MMP2/MMP9, and increasing the expression of epithelial marker E-cadherin. Inhibits cell stemness through inhibition of HGF/Met pathway.	[54]
Sanguisorba officinalis	Ziyuglycoside II (3β-3-α-1-arabinopyranosyloxy-19-hydroxyurs-12-en-28-oicacid)	In vitro	HepG2, SMMC-7721	Impedes cell cycle proliferation and causes apoptosis through cell cycle arrest at G0/G1. Suppression of migration and invasion through downregulation of MMP2 and MMP9, while also inhibiting the EGFR/NF-kB pathway.	[55]
Brucea javanica	Brucea javanica oil	In vivo	N/A	Induced expression of tumor suppressors miRNA-29b and p53. Induced apoptosis through downregulation of anti-apoptotic Bcl-2 and upregulation of pro-apoptotic Bax, Bac, cytosol cytochrome-c, cleaved caspase-3, cleaved caspase-9, and PARP.	[56]
Artemisia annua	Artemisinin	In vitro In vivo	SMMC-7721	Inhibit proliferation through blocking PI3K/AKT and mTOR signaling pathways. Induces apoptosis through downregulating anti-apoptotic proteins XIAP and survivin, and upregulating pro-apoptotic proteins cleaved caspase-3 and PARP. Inhibits invasive and migratory ability. Inhibits metastasis through increasing cell–cell adhesion.	[57,58]
Citrus bergamia	Eriocitrin, Neoeriocitrin, Naringin, Neohesperidin	In vitro	HepG2	Decreases proliferation through cell cycle arrest at G2 phase through upregulation of p53 and p21. Induces apoptosis through increased expression of pro-apoptotic genes Bcl-2, caspase 8, caspase 9, caspase 3, PARP, TNF receptor, NF-kB, and IkB. Downregulates anti-apoptotic genes Birc-8 and BCL212.	[59,60]
Scutellariae radix	Oroxylin A	In vitro	HepG2	Reduces metabolic capability of cancer cells under hypoxic conditions through reducing the generation of lactate and glucose; inhibits expression of metabolic regulator HIF-1a. Causes differentiation of cancer cells through activation of HNF-4 a, thereby decreasing metastatic potential.	[61,62]
Various fruits and vegetables such as Kale, berries, apples	Quercetin	In vitro In vivo	HepG2	Induces apoptosis through upregulation of p53 and Bax. Inhibits glycolysis through reduction of key glycolysis enzyme HK-2, as well as by reducing expression of phosphorylated mTOR and Akt.	[63,64,65,66,67]
Various fruits and vegetables such as strawberries, apples, cucumbers, grapes, onions	Fisetin	In vitro	HepG2,	Inhibits proliferation through cell cycle arrest. Induces apoptosis and necroptosis through increased expression of TNFalpha, Bax, caspase-3, and PARP, and through increased expression of RIPK1, pRIPK1, RIPK3, pRIPK3, and MLKL; decreased expression of NF-kB, pNF-kB, and pIKkB.	[68]
Licorice plant	Glabridin	In vitro	HepG2, Huh-7, MHCC97H Sk-Hep-1	Decreases stemness through inhibition of TGF-beta/SMAD2 signaling pathway. Decreases invasive capabilities through downregulation of MMP-9 and MMP-1. Suppresses tumor formation in xenograft model.	[69,70]
Soybeans	Genistein	In vitro	Bel-7402 Hep3B	Inhibits tumor growth through cell cycle arrest at G0/G1 and G2/M. Promotes apoptosis and increases phosphor-AMPK expression.	[71,72]
Various fruits and vegetables	Luteolin	In vitro In vivo	HepG2, Huh7	Causes cytotoxicity through increase production of ROS and release of cytochrome-c. Impedes growth through increased expression of miR-6809-5p, which blocks activation of growth cell signaling regulator FLOT1.	[73,74]
Fagopyrum tataricum	Tatariside F	In vitro In vivo	H22	Induces apoptosis through upregulation of p53 and Bax, and through downregulation of Bcl-2. Inhibits tumor growth in vivo.	[75]
Pulicaria jaubertii	Pulicaria jaubertii 1	In vitro	HepG2	Induces apoptosis through activation of p53, as well as through inhibiting K-Ras/B-Raf protein expression	[76]
Jujube Leaf	Jujube Leaf Green Tea Extract	In vitro	HepG2	Inhibits proliferation and induces apoptosis through activation of AMPK	[77]
Camellia sinensis	Epigallocatechin-3-gallate	In vitro In vivo	HepG2, Hep3B, Huh7, SMMC7721, sk-hep1, hcc-lm3	Inhibits proliferation through inhibiting ERalpha36, and through inhibiting PI3K/Akt and MAPK/ERK pathways. Induces apoptosis through caspase 3 activation and induction of the ERα36-EGFR-Her-2 feedback loop.	[78,79,80,81,82,83,84,85,86,87,88]
Blueberries, grapes, and tree wood	Pterostilbene	In vitro In vivo	HepG2	Inhibits migration, invasion, and proliferation through downregulation of MMP-9 and through suppression of TPA-induced PI3K-AKT-NF-κB signaling. Inhibits in-vivo metastasis.	[89,90]
Grapes, peanuts, and pines	Resveratrol	In vitro In vivo	SMMC-7721, Bel-7402, HepG2	Inhibits call growth through inhibition of metabolic phenotypes that support anaerobic growth.	[32,91,92,93,94,95,96,97,98,99]
Garcinia mangostana	Mangostanaxanthone V	In vitro	HepG2	Inhibits proliferation through cell cycle arrest at G0/G1	[100]
	Mangostanaxanthone VI	In vitro	HepG2	Inhibits proliferation through cell cycle arrest at G2/M	[100]
	Alpha-mangostin	In vitro	HepG2	Sensitizes cells to anoikis through stimulation of pro-apoptotic mechanisms such as induction of caspase-9, capsase-8, and caspase-3, and through downregulation of anti-apoptotic proteins. Inhibits migration through inhibition of MMP-2 and MMP-9, and through suppression of AKT and ERK signaling pathways.	[101]
Coffee beans, cacao beans, green tea leaves	Caffeine	In vitro In vivo	HCC Cell line	Works in conjunction with 5-fluorouracil to decrease proliferation and induce apoptosis through increased intracellular ROS production.	[41]
Rhizoma coptidis	Berberine	In vitro	HepG2	Reduces proliferation and induces apoptosis through suppression via p65 of the NF-kB pathway.	[102]
Nelumbo nucifera	Neferine	In vitro	Hep3B	Induces apoptosis through downregulation of cell cycle markers and through induction of ER stress.	[103]
Garcinia	Gambogic acid	In vitro	HepG2, SMMC-7721	Reduces proliferation and induces necrosis and apoptosis through induction of oxidative stress.	[104,105,106]
Poncirus trifoliata	*Poncirus fructus*	In vitro	Hep3B, Huh7	Inhibits proliferation and induces apoptosis inducing loss of mitochondrial membrane potential and high intracellular ROS levels. Inhibits migratory ability through downregulation of mesenchymal markers and upregulation of epithelial marker E-cadherin; reduces MMP-2 and MMP-9.	[107]
Alpinia galangal	1′-Acetoxychavicol acetate	In vitro	HepG2	Induces apoptosis through upregulation of ROS and NADPH oxidase.	[108]
White Birch Trees	Betulinic acid	In vitro In vivo	HepG2	Induces apoptosis through the mitochondrial pathway.	[109]
Panax ginseng	Ginsenoside Rh2	In vitro In vivo	HepG2	Incudes apoptosis through the mitochondrial pathway.	[109]
Huanglian decoction	*Coptidis Rhizoma,* *Zingiberis Rhizoma,* *Folium Artemisiae Argyi,* *Mume Fructus*	In vitro In vivo		Inhibits migration and invasion through inducing G2/M cell cycle arrest. Induces apoptosis through downregulation of CCNB1 genes, which results in activation of p53 pathway through upregulation of Bax, caspase-3, caspase-9, p21, and p53. Suppresses tumor growth and angiogenesis in xenograft mouse model.	[110,111,112]

**Table 2:** Phytochemicals used in clinical trials for HCC treatment.

Plant Sources	Treatment	Traditional Therapy	Clinical Trial Phase	Proposed Role in HCC	Dosage	Route of Administration	Conclusions	References
*Basidiomycotina*	Active hexose correlated compound (AHCC)	Resection	Prospective cohort study	Enhances natural killer cell activity	3.0 g/day	Oral	AHCC improved postoperative HCC prognosis.	[113]
*Viscum album* L. (mistletoe)	Fraxini-2	None	Phase II	Cytotoxicity against tumor cells and stimulation of immune cells	Two 10,000 ng/mL ampoules given once, weekly	Subcutaneously	Viscum Fraxini-2 has anti-tumor activity and a low toxicity profile in HCC.	[114]
*Panax ginseng*	Ginsenoside	Transcatheter arterial chemoembolization (TACE)	Randomized controlled study	Downregulating hypoxia-induced VEGF expression, inhibiting proliferation and invasion, promoting apoptosis	TACE (7 days before ginsenoside): Oxaliplatin (75 mg/m^2^), 5-fluorouracil (500 mg/m^2^), epirubicin (30–50 mg/m^2^), given until it stayed in the vessels for more than 10 heartbeats, ginsenoside: 20 mg twice a day	Oral	The TACE and ginsenoside Rg3 combination may prolong overall survival compared to TACE alone.	[115]
*Trametes robiniophila Murr* mushrooms (Huaier granule)	Huaier granule + Fufang Banmao capsule + Jinlong capsule + Kanglixin capsule + Ganfule capsule	None	Retrospective cohort	Inducing intrinsic and extrinsic apoptotic pathways through p38 MAPK	Fufang Banmao capsule (0.0175 mg/g), Huaier granule (71.5 mg/g protein content), Jinlong capsule (31.2% active principle), Kanglixin capsule (0.562 mg/g to 3.874 mg/g), Ganfule capsule (25% active principle)	Oral	Traditional Chinese medicines may prolong median survival and overall survival in HCC patients.	[116]
*Epimedii herba*	Icaritin	None	Phase I	Immune-modulation activity through IL-6/Jak2/Stat3 pathways, natural killer cells, T cells, IFN-γ, and myeloid-derived suppressor cells	600 mg or 800 mg twice a day	Oral	Icaritin’s immune-modulation activities were correlated with safety profiles and preliminary survival benefits.	[117]
Camptotheca acuminata	Irinotecan	Patients with one prior chemotherapy program were allowed None	Phase II Phase I	DNA synthesis interference	125 mg/m^2^ weekly Starting at 7.5 mg/m^2^/day with an increase of 2.5 mg/m^2^/day	Not specified Percutaneous catheterization reservoir set in a subcutaneous pocket	Irinotecan showed modest activity on HCC with a substantial toxicity in patients. Intra-arterial infusion of Irinotecan had no major adverse events from the compound but had management concerns of the intra-arterial device.	[118] [119]
Ginseng, atractylodes, Tuckahoe, licorice root, *radix bupleuri,* yam, *cortex moutan, salvia miltiorrhiza*, turmeric, *rhizome zehoariae*	Jianpi Huayu Therapy	Hepatectomy	Randomized clinical study	Improve immune function	Ginseng 20 g, atractylodes 15 g, Tuckahoe 15 g, licorice root 6 g, radix bupleuri 15 g, yam 12 g, cortex moutan 10 g, salvia miltiorrhiza 15 g, turmeric 10 g, rhizoma zedoariae 10 g, given once daily, 3 days after hepatectomy	Not specified	Jianpi Huayu therapy and hepatectomy together decreased post-op reoccurrence and metastasis, while disease-free survival and overall survival improved.	[120]
Pilose Asiabell root, Largehead Atractylodes rhizome, Pinella tuber, Hawthorn fruit, Common Yam rhizome, Fu-ling, Areca peel, Licorice root, Virgate Wormwood herb. Pilose Asiabell root, Largehead Atractylodes rhizome, Pinella tuber, Hawthorn fruit, Common Yam rhizome, Fu-ling, Areca peel, Licorice root, Virgate Wormwood herb	Jianpi Ligan decoction: *Codonopsis pilosula, Rhizoma Atractylodis macrocephala, Rhizoma Pinelliae, Fructus Crataegi, Rhizoma Diosscoreae, Poria cocos, Pericarpium Arecae, Radix Glycyrrhizae, Herba Artemisia scoparia* Jianpi Ligan decoction: *Codonopsis pilosula, Rhizoma Atractylodis macrocephala, Rhizoma Pinelliae, Fructus Crataegi, Rhizoma Diosscoreae, Poria cocos, Pericarpium Arecae, Radix Glycyrrhizae, Herba Artemisia scoparia*	Transcatheter arterial chemoembolization (TACE) Radiofrequency Ablation (RFA)	Retrospective clinical study Retrospective clinical study	Tonifies the spleen, tonifies the stomach, improves digestion, relieves constipation, promote urination, decrease ascites, liver detoxification, clears jaundice Tonifies the spleen, tonifies the stomach, improves digestion, relieves constipation, promote urination, decrease ascites, liver detoxification, clears jaundice	TACE: 5-fluorouracil (1000 mg/m^2^), cisplatin (80 mg/m^2^), and Jianpi Ligan decoction (given on same day as TACE): Radix Codonopsis (20 g), Rhizoma Atractylodis macrocephala (10 g), Poria cocos (15 g), Radix Glycyrrhizae (5 g), Rhizoma Diosscoreae (15 g), Rhizoma Pinelliae (10 g), Fructus Crataegi (15 g), Semen Nelumbinis (20 g), Herba Artemisia scoparia (50 g), Pericarpium Arecae (25 g) Jianpi Ligan decoction 100 mL orally once per day, 30 min after meals composed of: Pilose Asiabell Root (20 g), Largehead Atractylodes Rhizome (10 g), Fu-ling (15 g), Liquorice Root (5 g), Common Yam Rhizome (15 g), Pinellia Tuber (10 g), Hawthorn Fruit (15 g), Semen Nelumbinis (20 g), Virgate Wormwood Herb (50 g), Areca Peel (25 g)	Not specified Oral	TACE and the Jianpi Ligan decoction combined showed a side effect reduction and improved long-term survival in TACE treated, unresectable HCC patients. RFA and the Jianpi Ligan decoction combined showed a significantly higher rate of treatment success, and a significantly higher 3-year overall survival when compared to controls.	[121] [122]
Root of *Salvia chinensis,* root of *Actinidia valvata, Cremastra appendiculata* Root of *Salvia chinensis,* root of *Actinidia valvata, Cremastra appendiculate,* root of *Salvia chinensis,* ruber of *Pseudobulbus cremastrae*	Jiedu granule Jiedu granule + root of *Salvia chinensis* Benth + tuber of *Pseudobulbus cremastrae* seu Pleiones + gizzard of *Gallus domesticus* Brison + extract from *Buro gargarizans* Cantor	Transcatheter arterial chemoembolization (TACE), and Gamma Knife radiosurgery (GKR) Transcatheter arterial chemoembolization (TACE)	Retrospective clinical study Randomized controlled study	Exact mechanism is unclear Inhibition of tumor cell growth, induction of apoptosis, suppression of angiogenesis, enhanced immune function	TACE: lipiodol (10–20 mL) and pirarubicin (30 mg) assessed by radiography, Gamma Knife radiosurgery (GKR) isodose of 55%, 5.9 g Jiedu granule twice a day after 1 week of TACE Traditional herbal medicine (THM) using Cinobufacini (50 mL) taken once daily, Jiedu granule (4.5 g) taken twice a day, and TACE: pirarubicin (10 mg), mitomycin (10 mg), iodipin (2 to 5 mL)	Oral Cinobufacini (intravenous drip), Jiedu granule (orally)	Jiedu granule combined with TACE and Gamma Knife Radiosurgery is safe for HCC patients with portal vein tumor thrombosis. Jiedu granule promoted better prognosis as well. Traditional herbal medicine was superior to TACE for preventing disease recurrence and prolonging overall survival in small HCC.	[123] [124]
Poppy seed oil Poppy seed oil Poppy seed oil	Intra-arterial iodine-131 labeled Lipiodol Intra-arterial 131I-labeled Lipiodol Chemoembolization with Lobaplatin mixed with Lipiodol (iodized oil)	Resection None Orthotopic liver transplantation	Prospective randomized trials Retrospective controlled clinical trial Retrospective controlled clinical trial	Eradicates microscopic tumor loci Eradicates microscopic tumor foci Induces tumor ischemia, and allows local high concentration of chemotherapy	2 mL of iodine-131 lipiodol at 1850 MBq per treatment 5 mL volume of iodine-131 lipiodol at a dose of 2.2 GBq Lobaplatin (50 mg/m^2^), iodized oil (ranging from 2 mL to 5 mL)	Artery cannulation Intra-arterially Subcutaneous pump and catheter	Intra-arterial iodine-131 labeled lipiodol after resection decreased recurrence rate and increased disease-free survival and overall survival in HCC patients. Intra-arterial iodine-131 injection was shown to be safe and had benefits for overall survival in patients with advanced HCC. Lobaplatin-based chemoembolization can improve overall survival and elicit effect tumor response for patients with unresectable HCC.	[125] [126] [127]
Bark of Pacific Yew tree	Paclitaxel	None	Phase I	Microtubule inhibition	Given as 1 h infusions on selected days and escalating doses of: 70 mg/m^2^, 80 mg/m^2^, 90 mg/m^2^, and 100 mg/m^2^	Intravenously	One patient achieved partial remission and 9 other patients had prolonged stable disease. There was also an improvement in tumor-related symptoms.	[128]
Pseudostarwort root, milkvetch root, poria, white atratylodes tuber, rehmannia root, dendrobium, white peony root, Chinese angelica root, aldenlandia herb, Chinese lobelia, wild ginger, licorice root	Shenqi mixture	Microwave coagulation	Retrospective clinical study	Lymphocyte stimulation, macrophage stimulation, humoral immunity enhancement, cellular immunity enhancement, reduce glutamic pyruvic transaminases, enhancement of reticuloendothelial system, hepatoprotective, anti-inflammatory	Microwave coagulation: 60 W output power or 800 seconds, Shenqi mixture (given the day after microwave coagulation): 20 mL (20 g of crude compounds), 3 times a day	Oral	The Shenqi mixture and microwave coagulation killed tumor cells and prevented recurrence as well as enhanced cellular immunity without adverse reactions.	[129]
Root of *Marsdenia tenacissima*	Xiaoaiping injection	None	Randomized controlled study	Prevent cancer cell proliferation, improve proliferation f T and B lymphocytes	40 mL of Xiaoaiping injection	Intravenous drip	Xiaoaiping injection enhanced quality of life in advanced HCC patients, improved immunity, and extended progression-free survival.	[130]
*Coriolus versicolor* mushrooms	Polysaccharide peptide isolated from Yunzhi/kawaratake	None	Randomized controlled study	Inhibit IL-17F release, maintain prolactin levels, increase TRAIL R1	*Coriolus versicolor* (2.4 g/day)	Suggested as intravenous	No difference in time to progression when using *Coriolus versicolor* (Yunzhi) but HCC patients had better quality of life compared to placebo. *Coriolus versicolor* (Yunzhi) should be further explored for palliative care.	[131]
